# A novel mitochondrial pyruvate carrier inhibitor drives stem cell-like memory CAR T cell generation and enhances antitumor efficacy

**DOI:** 10.1016/j.omton.2024.200897

**Published:** 2024-10-18

**Authors:** Mathias Wenes, Anouk Lepez, Vladimir Arinkin, Kinsey Maundrell, Orsolya Barabas, Federico Simonetta, Valérie Dutoit, Pedro Romero, Jean-Claude Martinou, Denis Migliorini

**Affiliations:** 1 AGORA Cancer Research Center, 1005 Lausanne, Switzerland; 2 Swiss Cancer Center Léman, Geneva, Lausanne, Switzerland; 3 Center for Translational Research in Onco-Hematology, University of Geneva, 1206 Geneva, Switzerland; 4 MPC Therapeutics, 1206 Geneva, Switzerland; 5 Department of Molecular and Cellular Biology, University of Geneva, 1206 Geneva, Switzerland; 6 Division of Hematology, Department of Oncology, Geneva University Hospitals (HUG), 1206 Geneva, Switzerland; 7 Faculty of Biology and Medicine, University of Lausanne, 1015 Lausanne, Switzerland; 8 Novigenix SA, 1066 Epalinges, Switzerland; 9 Department of Oncology, Geneva University Hospitals (HUG), 1206 Geneva, Switzerland

**Keywords:** MT: Regular Issue, CAR T cell therapy, mitochondrial pyruvate carrier, immunometabolism, memory T cell differentiation, CAR T cell manufacture

## Abstract

Adoptive cell transfer with chimeric antigen receptor (CAR)-expressing T cells can induce remarkable complete responses in cancer patients. Therapeutic success has been correlated with central and stem cell-like memory T cell subsets in the infusion product, which are better able to drive efficient CAR T cell *in vivo* expansion and long-term persistence. We previously reported that inhibition of the mitochondrial pyruvate carrier (MPC) during mouse CAR T cell culture induces a memory phenotype and enhances antitumor efficacy against melanoma. Here, we use a novel MPC inhibitor, MITO-66, which robustly induces a stem cell-like memory phenotype in CD19-CAR T cells generated from healthy donors and patients with relapsed/refractory B cell malignancies. MITO-66-conditioned CAR T cells were superior in controlling human pre-B cell acute lymphoblastic leukemia in mice. Following adoptive cell transfer, MITO-66-conditioned CAR T cells maintained a memory phenotype and protected cured mice against tumor rechallenge. Furthermore, in an *in vivo* B cell leukemia stress model, CD19-CAR T cells generated in the presence of MITO-66 largely outperformed clinical-stage AKT and PI-3Kδ inhibitors. Thus, we provide compelling preclinical evidence that MPC inhibition with MITO-66 during CAR T cell manufacturing dramatically enhances their antitumor efficacy, thereby paving the way to clinical translation.

## Graphical abstract

**Figure:**
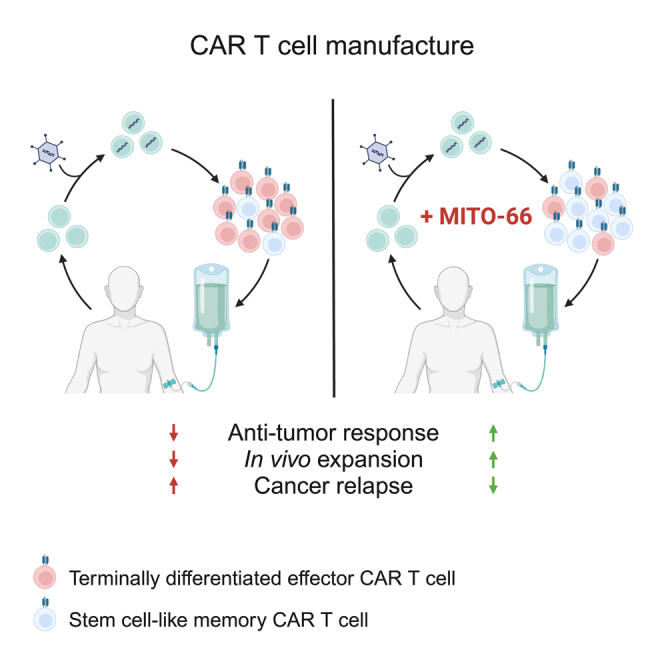

## Teaser

Wenes and colleagues show that manufacturing anti-CD19 CAR T cells with a novel mitochondrial pyruvate carrier inhibitor, MITO-66, induces a stem cell-like memory T cell phenotype. MITO-66-conditioned CAR T cells are superior in controlling human B-ALL in mice and prevent relapse.

## Introduction

Adoptive T cell transfer with either *ex vivo* expanded tumor-infiltrating lymphocytes or T cells genetically modified to express a T cell receptor (TCR T) or a chimeric antigen receptor (CAR T), has shown remarkable therapeutic efficacy in metastatic melanoma, neuroblastoma, and certain types of blood malignancies.^1^^,^^2^^,^^3^^,^^4^ However, a large fraction of patients fail to achieve durable complete remission and eventually relapse. An important factor in this suboptimal clinical response is the terminal differentiation and exhaustion of adoptively transferred cells.^5^^,^^6^ Instead, it is well established that enhanced proportions of less differentiated central memory (T_CM_) or stem cell-like memory (T_SCM_)/naive T cells either at leukapheresis or in the drug product correlate positively with a durable response due to increased *in vivo* expansion and long-term persistence of therapeutic T cells.^7^^,^^8^^,^^9^^,^^10^^,^^11^^,^^12^

T cell activation and differentiation is closely linked to cellular metabolism. Activated effector T cells critically depend on glycolysis, but also induce mitochondrial metabolic pathways such as oxidative and reductive metabolism of glutamine.^13^^,^^14^^,^^15^ In contrast, memory T cells depend more on mitochondrial metabolism and oxidative phosphorylation, and interference with these metabolic processes directly influences T cell differentiation.^16^^,^^17^^,^^18^ We previously found that genetic and pharmacological inhibition of the mitochondrial pyruvate carrier (MPC) favored memory T cell differentiation through a metabolic-epigenetic axis. Consequently, murine CAR T cells produced in the presence of the small-molecule MPC inhibitor UK-5099 strongly suppressed melanoma tumor growth.^19^

The MPC is a heterodimer consisting of two proteins, MPC1 and MPC2 located in the inner mitochondrial membrane. Although its molecular identity was only revealed about a decade ago,^20^^,^^21^ Halestrap et al. in 1975 had already described the ability of UK-5099 to potently inhibit pyruvate-dependent oxygen consumption by rat heart mitochondria.^22^ UK-5099 likely inhibits the MPC by covalently binding a cysteine residue on MPC2,^23^ although this mode of action has been questioned.^24^ Only recently, novel UK-5099 analogs were developed as MPC inhibitors for topical treatment of hair loss.^25^ However, like UK-5099, the novel MPC inhibitors all possess a Michael acceptor unit, which has long been controversial in drug design due to fear of off-target effects and potential toxicity.^26^ Thus, there remains a need to develop alternative MPC inhibitors with a good safety profile that would allow their use in therapeutic contexts.

Here, we describe a novel and specific MPC inhibitor, MITO-66, which robustly induces a T_SCM_ phenotype in human CAR T cells from both healthy donors and cancer patients. MITO-66-conditioned CAR T cells displayed impressive anti-leukemic efficacy and protected mice against leukemia relapse. Furthermore, MPC inhibition was superior to other state-of-the-art small molecules that induce a memory T cell phenotype, thus promoting its use in clinical CAR T cell manufacturing protocols.

## Results

### MITO-66 is a novel small-molecule inhibitor of the MPC

To screen for novel MPC inhibitors, we used our previously developed bioluminescence resonance energy transfer (BRET)-based biosensor, RESPYR, in which MPC1 is fused to the donor group RLuc8 (a variant of Renilla luciferase) and MPC2 is fused to the acceptor group Venus (a variant of yellow fluorescent protein).^27^ In this system, conformational changes due to substrate or inhibitor binding alter the proximity between the termini and will result in a measurable change in energy transfer and an increase in luminescence. We thus screened a library of ∼70,000 small molecules, and selected a top hit (MITO-1, Figure S1A). The inhibitory effect of MITO-1 was further confirmed by its capacity to inhibit oxygen consumption of HeLa cells, in a Seahorse assay in which the only carbon source available was pyruvate, with an IC_50_ of 690 nM (Figure S1B). Medicinal chemistry performed on this molecule resulted in the synthesis of a novel and more potent compound, which we called MITO-66, having a Seahorse IC_50_ of 119 nM (Figure S1C), and an RESPYR IC_50_ of 105 nM (Figure S1D). To test whether MITO-66 was inhibiting pyruvate import through direct binding to the MPC, we purified the MPC1/MPC2 heterodimer and performed a thermoshift assay (Figure S1E). The results showed a shift in the melting temperature of the unbound MPC1/MPC2 heterodimer from 38.2°C to 56.1°C in the presence of MITO-66, leading to a Kd estimation of 320 nM (Figure S1F). Overall, these results support a direct binding of MITO-66 to the MPC. Importantly, DEREK^28^ studies predicted no mutagenic activity of the molecule.

Thus, MITO-66 is a novel and potent MPC inhibitor.

### MITO-66 induces a stem cell-like memory phenotype in CAR T cells

**Figure 1:**
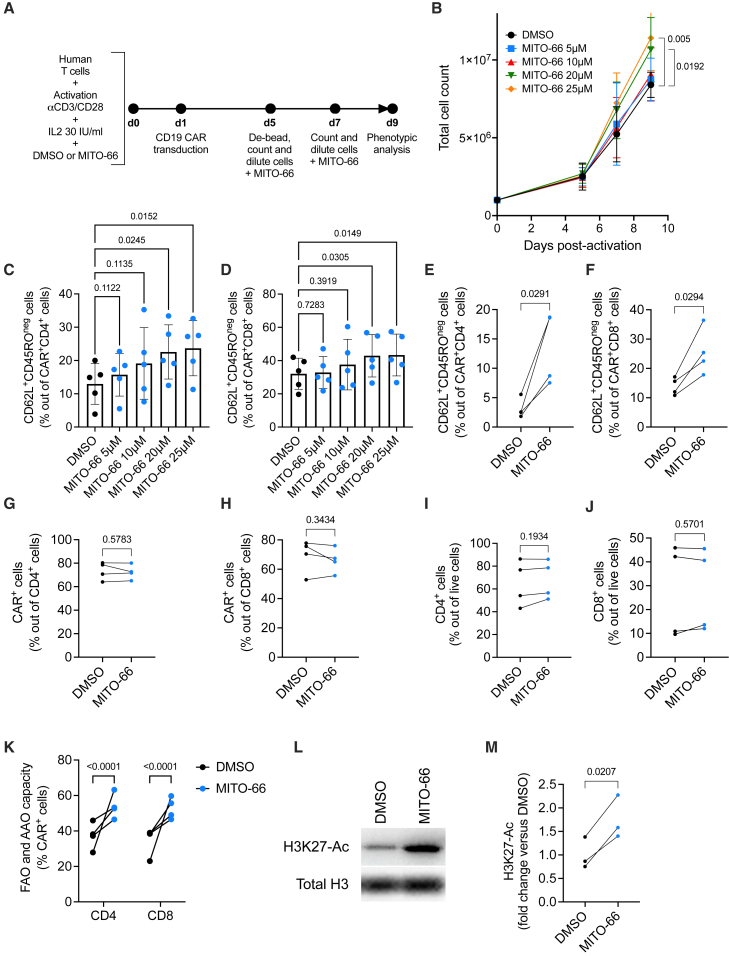
MITO-66 induces a stem cell-like memory phenotype in CAR T cells (A) Experimental scheme depicting CD19-CAR T cell generation in the presence of MITO-66. (B) T cell expansion measured by machine-assisted trypan blue-based cell counting on days 5, 7, and 9 post-activation (five donors, pooled data from three independent experiments). (C and D) CD62L-positive, CD45RO-negative stem cell-like memory T cells out of CD4 (C) or CD8 (D) CAR T cells at day 9 post-activation (five donors, pooled data from three independent experiments). (E and F) CD62L-positive, CD45RO-negative stem cell-like memory T cells in the CD4 (E) or CD8 (F) CAR T populations at day 9 post-activation (four donors, pooled data from two independent experiments). (G and H) CAR-positive T cells in the CD4 (G) and CD8 (H) populations at day 9 post-activation (four donors, pooled data from two independent experiments). (I and J) CD4 (I) and CD8 (J) T cells out of total live cells at day 9 post-activation (four donors, pooled data from two independent experiments). (K) Fatty acid oxidation (FAO) and amino acid oxidation (AAO) measured by SCENITH in CD4 and CD8 CAR T cells at day 9 post-activation (four donors, pooled data from two independent experiments). (L and M) Representative western blot (L) and quantification of H3K27 acetylation, normalized by total histone H3 protein levels in T cells at day 9 post-activation (three donors, pooled data from two independent experiments). Data are represented as mean ± standard deviation (SD). Statistics are based on one-way ANOVA (B–D), on paired, two-tailed Student’s t test (E–J and M), or two-way ANOVA (K). See also Figure S1.

We next determined the effect of MITO-66 on axi-cel/brexu-cel-like CAR T cells, which are FDA-approved for the treatment of large B cell lymphoma, acute lymphoblastic leukemia, and mantle cell lymphoma. These CAR T cells express second-generation CAR constructs targeting CD19, contain an intracellular CD28 costimulatory domain and are manufactured with IL-2.^29^^,^^30^ For our experiments, CD19-28ζ CAR T cells were generated from T cells enriched from the peripheral blood of healthy donors in the presence of different concentrations of MITO-66, or DMSO alone as a control (Figure 1A). MITO-66 slightly increased dose dependently the total yield of the T cell product by the end of the culture, 9 days post-activation (Figure 1B). Importantly, MITO-66 also dose dependently increased the proportion of stem cell-like memory CD45RO-negative, CD62L-positive CD4, and CD8 CAR T cells (Figures 1C and 1D), most potently and significantly at 25 μM. We thus selected 25 μM MITO-66 for subsequent testing. We confirmed induction of a T_SCM_ phenotype in four additional donors, while CAR transduction efficiency and the proportions of CD4 and CD8 T cells were not affected (Figures 1E–1J). Interestingly, MITO-66 increased the capacity of the CAR T cells to engage into fatty acid and amino acid oxidation, as compensation for a loss of mitochondrial pyruvate import (Figure 1K), similar to what we have shown in murine T cells following culture with UK-5099.^19^ Furthermore, increased acetylation of lysine residue 25 on histone H3, a characteristic of memory T cell differentiation, was observed upon CAR T cell manufacture with MITO-66 (Figures 1L and 1M). Finally, after performing a minimal wash-out procedure, consisting of two centrifugation-resuspension steps, only trace amounts of MITO-66 were detected in the cell pellet (9.14 ± 4.14 ng per 10^8^ cells), suggesting negligible patient exposure.

Thus, we conclude that MPC inhibition by MITO-66 during CD19-CAR T cell generation induces a T_SCM_ phenotype with no negative impact on yield and transduction efficiency.

### MITO-66 conditioning enhances CAR T cell expansion and cytotoxicity

**Figure 2:**
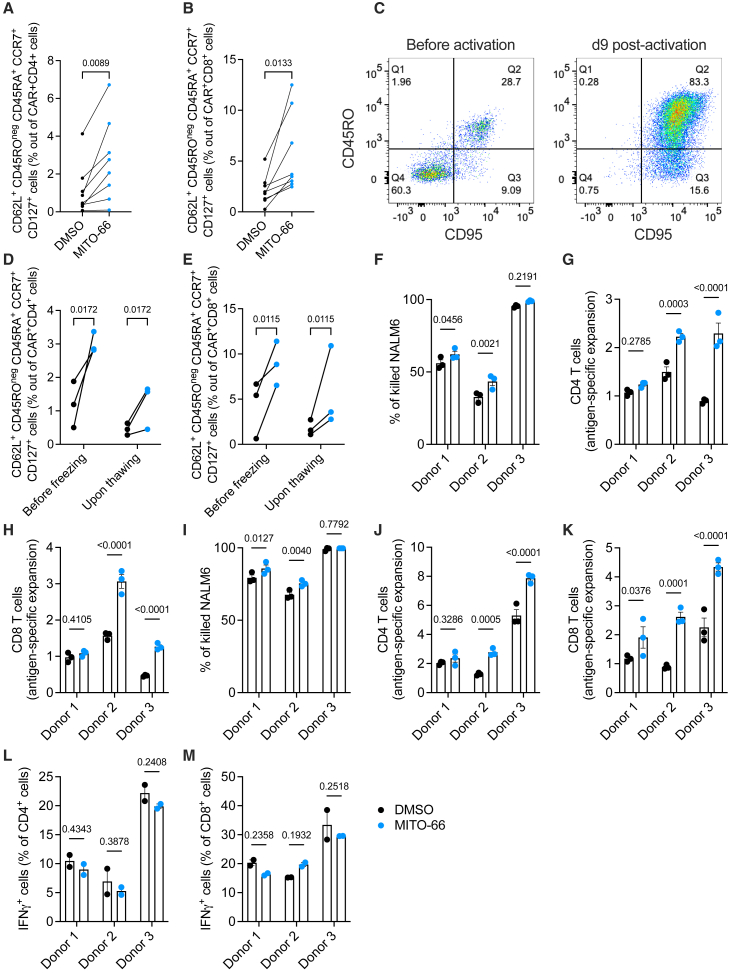
MITO-66 conditioning enhances CAR T cell expansion and cytotoxicity (A and B) CD45RO-negative, CD62L/CD45RA/CCR7/CD127-positive stem cell-like memory T cells in the CD4 (A) or CD8 (B) CAR T populations at day 9 post-activation (six donors, pooled data from four independent experiments). (C) Representative flow cytometry plots depicting CD45RO and CD95 in isolated T cells from a healthy donor before (left) and 9 days after (right) activation with anti-CD3:CD28 beads. (D and E) CD45RO-negative, CD62L/CD45RA/CCR7/CD127-positive stem cell-like memory T cells in the CD4 (D) or CD8 (E) CAR T populations at day 9 post-activation just before freezing or right after thawing (three donors, CAR T cell manufactured during three independent experiments). (F–M) CAR T cells were thawed and immediately exposed to NALM6 cells at a 1:2 effector-to-target ratio. Tumor cell killing (F and I), T cell expansion (G, H, J, and K), and cytokine expression (L and M) were analyzed by FACS after 72 h (F–H, L, and M) or 7 days (I–K) of co-culture. Data are represented as mean ± SD. Statistics are based on paired, two-tailed Student’s t test (A and B) or on one-way ANOVA (D–M).

Although CD45RO^neg^ and CD62L^+^ cell surface markers are sufficient to identify naive and stem cell-like memory T cells, these cells are ideally further characterized by expression of CD45RA, CCR7, and CD127, while the expression of CD95 differentiates T_SCM_ from CD95-negative naive T cells.^31^ We confirmed that MITO-66 induced T_SCM_ cells when this population was identified more stringently by including the above-mentioned markers (Figures 2A and 2B). All cells at the end of the culture were CD95 positive (Figure 2C), suggesting that the CAR T cell population identified by CD45RO^neg^ and CD62L^+^, complemented or not with CD45RA^+^, CCR7^+^, and CD127^+^, are T_SCM_ cells.

We next functionally characterized MITO-66-conditioned CAR T cells in a stringent *in vitro* killing assay. Frozen CAR T cells were thawed and immediately co-cultured with CD19-expressing NALM6 leukemia cells at a 1:2 effector-to-target ratio. The thawing process maintained the T_SCM_ phenotype induced by MITO-66 (Figures 2D and 2E) but resulted in an inefficient killing in two out of three donors after 72 h of co-culture (Figure 2F). However, in those two donors, MITO-66 conditioning significantly enhanced the killing of NALM6 cells at 72 h and 7 days of co-culture (Figures 2F and 2I). Both CD4 and CD8 CAR T cell expansion was enhanced after 72 h and 7 days of co-culture (Figures 2G, 2H, 2J, and 2K). Surprisingly, no difference in cellular IFN-γ production was observed after 72 h (Figures 2L and 2M).

In conclusion, MITO-66 induces a stem cell-like memory phenotype in CAR T cells, which is maintained upon a freeze-thaw cycle and results in superior tumor cell killing and CAR T cell expansion.

### MITO-66 conditioning during CAR T manufacturing enhances antitumor efficacy

**Figure 3:**
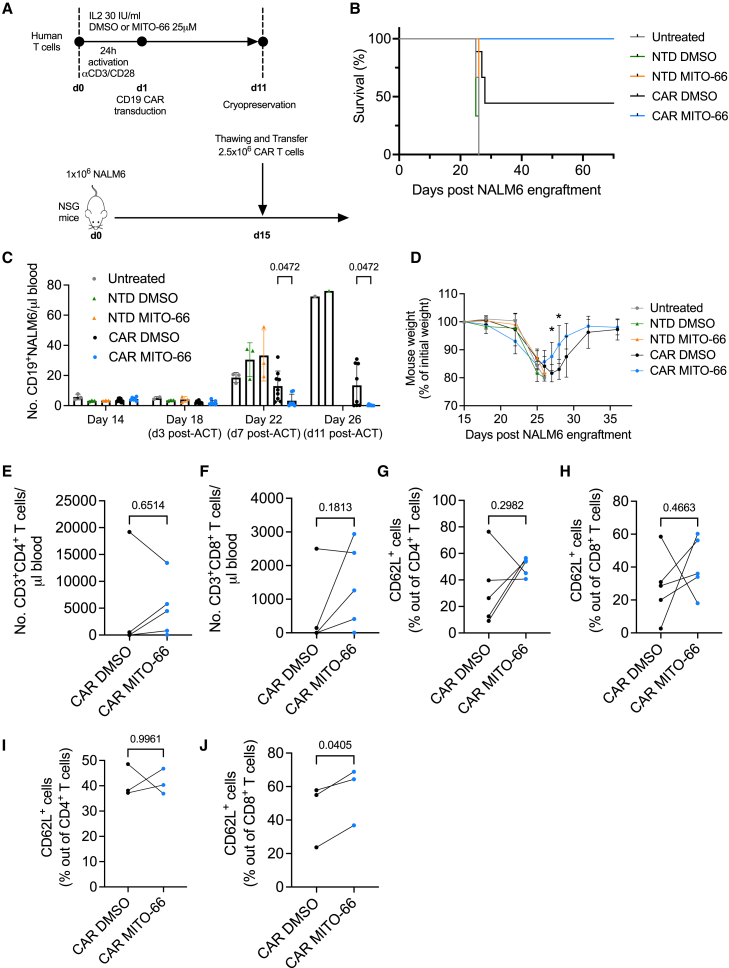
MITO-66 conditioning during CAR T manufacturing enhances antitumor efficacy (A) Experimental scheme depicting CD19-CAR T cell treatment of NALM6 leukemia-bearing mice. CAR T cells were prepared from five different donors during three independent manufacturing experiments and frozen at the end of the culture. At the time of ACT, the CAR T cells from all donors were thawed and administered to one or two recipient mice. (B) Survival of mice receiving no treatment, or non-transduced T cells (NTD) or CAR T cells manufactured with DMSO or MITO-66. (C) Number (No.) of CD19-positive NALM6 cells in the blood of mice analyzed by flow cytometry at the indicated time points. (D) Mouse weight expressed as percentage of the weight at the beginning of the experiment. (B and D) Uuntreated, NTD DMSO, and NTD MITO-66: *n* = 3 mice, CAR DMSO: *n* = 9 mice, CAR MITO-66: *n* = 7 mice. (E and F) Number of CD4 (E) or CD8 (F) T cells in the blood at 11 days post-ACT. (G–J) Percentage of CD62L-positive cells in CD4 or CD8 T cell populations at day 11 post-ACT (G and H) or day 33 post-ACT (I and J). (E–H) Four donors, pooled data from two independent experiments. (E–J) Data from mice receiving CAR T cells from the same donor were pooled and average was calculated, represented here is the paired analysis of five donors (E–H) or three donors in surviving mice (I and J). Data are represented as mean ± SD. Statistics are based on two-way ANOVA (C) or on unpaired two-tailed Student’s t test (D) or on paired, two-tailed Student’s t test (E–J). See also Figure S2.

Memory phenotype and mitochondrial fitness have been associated with superior antitumor function of CAR T cells.^9^^,^^32^^,^^33^ We tested the antitumor potential of MITO-66-conditioned CD19-CAR T cells following adoptive cell transfer in an NALM6-based mouse model of pre-B cell acute lymphoblastic leukemia. We opted for a stress-test model, in which 2.5 × 10^6^ CAR T cells were adoptively transferred in NOD-SCID-γc^−/−^ (NSG) mice 15 days following NALM6 engraftment, when the tumor burden is high (Figure 3A). Non-transduced T cells, either treated or untreated with MITO-66, had no effect on survival when compared with untreated mice, while DMSO-conditioned CAR T cell transfer prolonged survival and cured ∼44% of mice (Figure 3B). Remarkably, CD19-CAR T cells generated in the presence of MITO-66 cured 100% of mice (Figure 3B). NALM6 leukemic cells in the blood were efficiently suppressed by MITO-66-conditioned CAR T cells 7 days post-ACT, while the disease continued to progress in most mice treated with DMSO-conditioned CAR T cells (Figure 3C). Accordingly, MITO-66-conditioned CAR T cell-treated mice experienced less severe weight loss (Figure 3D). Eleven days post-ACT, almost no NALM6 leukemic cells were detected in the blood of cured mice (Figure 3C), while the number of CAR T cells generated with MITO-66 was, in four out of five donors, much higher in the blood compared with DMSO-conditioned CAR T cells (Figures 3E and 3F). When looking at individual *in vivo* CD4 and CD8 T cell expansion curves of the donor T cells that cured mice, we observed that MITO-66 conditioning resulted in either a higher peak expansion or anticipated expansion (Figure S2A). Phenotypically, CAR T cells generated with MITO-66 from four out of five donors displayed increased CD62L-positive memory T cell differentiation by day 11 post-ACT (Figures 3G and 3H), which was maintained in CD8 T cells at day 33 post-ACT (Figures 3I and 3J).

Thus, MITO-66 conditioning during preparation of CD19 CAR T cells induces a memory phenotype, which results in strongly enhanced antitumor efficacy and *in vivo* memory CAR T cell establishment.

### MITO-66-conditioned CAR T cells protect against cancer recurrence

**Figure 4:**
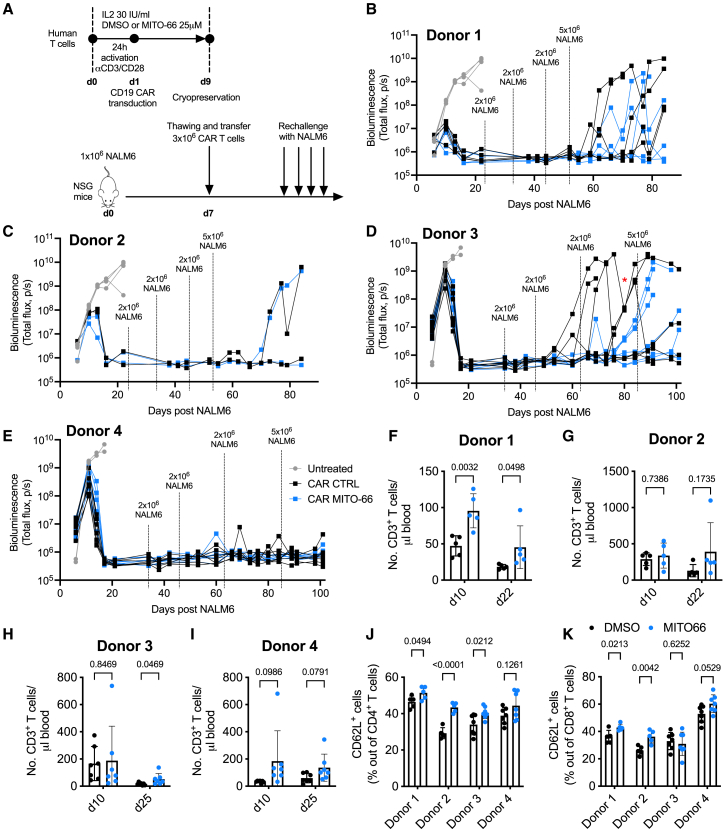
MITO-66-conditioned CAR T cells protect against cancer recurrence (A) Experimental scheme depicting CD19-CAR T cell treatment of NALM6 leukemia-bearing mice following *in vivo* rechallenge for three times with 2 × 10^6^ and finally 5 × 10^6^ NALM6 cells. CAR T cells were prepared from four different donors and performed in two independent experiments. CAR T cells from each donor were transferred in five to seven different NALM6-bearing mice. Throughout the experiment, some mice were lost because of graft-versus-host disease. (B–E) NALM6 tumor burden measured by bioluminescence at the indicated days post-initial NALM6 engraftment. (F–I) Number of transferred CD3 T cells in the blood of mice analyzed by flow cytometry at the indicated time points. (J and K) Percentage of CD62L-positive cells in the CD4 (E) or CD8 (F) T cell population in the blood at day 10 post-ACT. Data are represented as mean ± SD. Statistics are based on unpaired two-tailed Student’s t test (D) or two-way ANOVA (F–K).

Despite obtaining over 80% initial complete response rates in B-ALL following CAR T cell treatment, the majority of patients relapse within a year.^34^ Since MITO-66-conditioned CD19-CAR T cells maintain an increased memory phenotype *in vivo*, we wondered whether this would ensure protection against antigen-positive cancer recurrence. We thus generated CD19-CAR T cells with or without MITO-66, which were adoptively transferred in NSG mice at day 7 post-NALM6 engraftment, to obtain a higher number of mice cured with control CD19-CAR T cells (Figure 4A). Indeed, 15 days post-ACT, all mice receiving CAR T cells were cancer-free (Figure 4B). Interestingly, the first *in vivo* re-administrations of NALM6 cells did not induce cancer relapse, while the second and/or third rechallenge resulted in a strong cancer recurrence in DMSO-CAR-treated mice in two out of four donors but was much better controlled in MITO-66-CAR T cell-treated mice (Figures 4B–4D). One donor showed no difference (Figure 3C), while a fourth donor did not allow any cancer relapse throughout the entire experiment (Figure 4E). Interestingly, ACT at 7 days post-NALM6 resulted in a higher number of MITO-66-conditioned CAR T cells 22 days post-ACT (Figures 4F–4I). More importantly, we confirmed the increased proportion of CD62L-positive memory cells among the transferred CD4 and CD8 T cells (Figures 4J and 4K).

Therefore, MITO-66 conditioning during CAR T manufacturing induces a memory phenotype that persists *in vivo* and strongly reduces cancer recurrence.

### Benchmarking MITO-66 against other small molecules influencing memory differentiation

**Figure 5:**
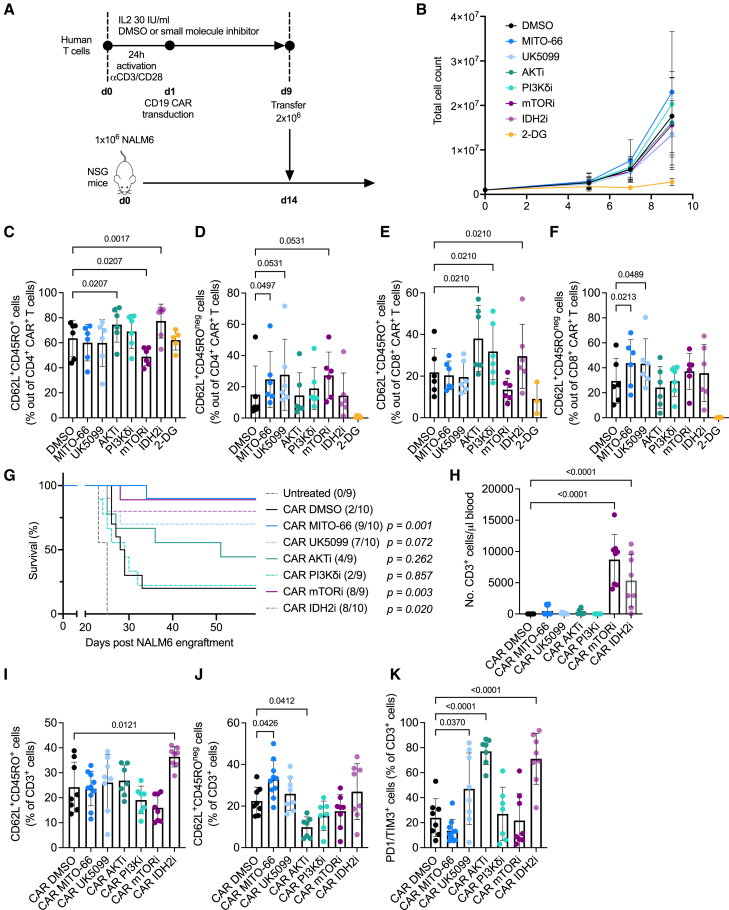
Benchmarking MITO-66 against other small molecules influencing memory differentiation (A) Experimental scheme depicting CD19-CAR T cell generation in the presence of different small-molecule inhibitors (MITO-66 and UK-5099 [25 μM], AKT-VIII [AKTi, 1 μM], idelalisib [PI3KδI, 200 nM], rapamycin [mTORi, 100 nM], enasidenib [IDH2i, 5 μM], and 2-deoxyglucose [2-DG, 2 mM], molecule concentrations were selected based on previous literature). (B) T cell expansion measured by machine-assisted trypan blue-based cell counting at day 5, 7, and 9 post-activation. (C–F) CD62L/CD45RO double-positive cells out of CD4 (C) or CD8 (E) CAR T cells or CD62L-positive, CD45RO-negative stem cell-like memory T cells out of CD4 (D) or CD8 (F) CAR T cells at day 9 post-activation. (B–F) Six donors, pooled data from four independent experiments. (G) Survival of mice receiving no treatment or inhibitor-conditioned CAR T cells. Surviving mice out of total treated mice are indicated in brackets. *p* values indicate statistical difference versus CAR DMSO. (H) Number (No.) of transferred CD3 T cells in the blood of mice analyzed by flow cytometry at day 11 post-ACT. (I–K) Percentage of CD62L/CD45RO double-positive cells (I), CD62L-positive, CD45RO-negative cells (J), or PD1/TIM3 double-positive cells (K) out of transferred CD3 T cells at day 11 post-ACT. (G–K) Three human donors into 9–10 total mice, pooled data from 2 independent experiments, only flow cytometry data with >20 events were used for phenotypic analysis. Data are represented as mean ± SD. Statistics are based on one-way ANOVA (C–F and H–K) or log rank test (G). See also Figure S3.

Previous studies on the induction of memory T cell differentiation have focused on small-molecule interference with components in the signaling cascade downstream of T cell activation, such as AKT, PI-3Kδ, and mTOR,^35^^,^^36^^,^^37^^,^^38^^,^^39^ using AKT-VIII (AKTi), idelalisib (PI3Kδi), or rapamycin (mTORi), respectively. Direct metabolic targeting has also been proposed, such as interference with glycolysis using 2-deoxyglucose (2-DG),^40^ and we have recently investigated the possibility of targeting either the MPC using UK-5099^19^ or targeting isocitrate dehydrogenase 2 (IDH2) using enasidenib (IDH2i).^15^ We therefore decided to perform a side-by-side comparison of MITO-66 versus the above-mentioned small-molecule inhibitors in CD19-CAR T cells (Figure 5A). 2-DG significantly affected CAR T cell proliferation, while inhibition of the other targets had no significant impact (Figure 5B). While AKT, PI-3Kδ, and IDH2 inhibition induced a CD45RO/CD62L double-positive T_CM_ phenotype, MPC inhibition with either MITO-66 or UK5099 induced a CD45RO-negative/CD62L-positive T_SCM_ phenotype (Figures 5C–5F). CAR T cells were frozen at day 9, then thawed and infused into NSG mice, 14 days post-NALM6 engraftment. Of note, the 2-DG-conditioned CAR T cells were not used for *in vivo* studies, because insufficient number of cells were generated. IDH2i-, mTORi-, and MPCi-conditioned CAR T cells most efficiently mediated long-term survival of leukemic mice, with MITO-66 performing slightly better than UK-5099 (Figure 5G). Surprisingly, PI3Kδi conditioning did not enhance antitumor efficacy, while AKTi conditioning partially enhanced mouse survival (Figure 5G). At day 11 post-ACT, mTORi- and IDH2i-conditioned CAR T cells were the most abundant in the blood, followed by MITO-66-conditioned CAR T cells (Figures 5H and S3A–S3D). Interestingly, the enhanced central memory phenotype was maintained in IDH2i-conditioned CAR T cells at 11 days post-ACT, while MITO-66-conditioned CAR T cells maintained an increased T_SCM_ phenotype (Figures 5I, 5J, S3E, S3F, S3H, and S3I). mTORi-, IDH2i-, and UK-5099-conditioned CAR T cells showed increased proportions of CD8 T_SCM_ cells (Figure S3I), while AKTi and PI3Kδi did not induce major phenotypic changes when compared with DMSO-conditioned CAR T cells. Finally, UK-5099-, AKTi-, and IDH2i-conditioned CAR T cells displayed higher levels of PD1/TIM3 double-positive T cells at day 11 post-ACT when compared with DMSO-conditioned CAR T cells, whereas this phenotype was not observed for MITO-66-conditioned CAR T cells (Figures 5K and S3G–S3J).

In conclusion, MPC inhibition using MITO-66 during preparation of the CAR T cells is among the best solutions to enhance CAR T cell antitumor efficacy and has the distinct advantage that the increased T_SCM_ phenotype following *in vivo* transfer is maintained.

### MITO-66 induces a T_SCM_ phenotype in CAR T cells from patients with B cell malignancies

**Figure 6:**
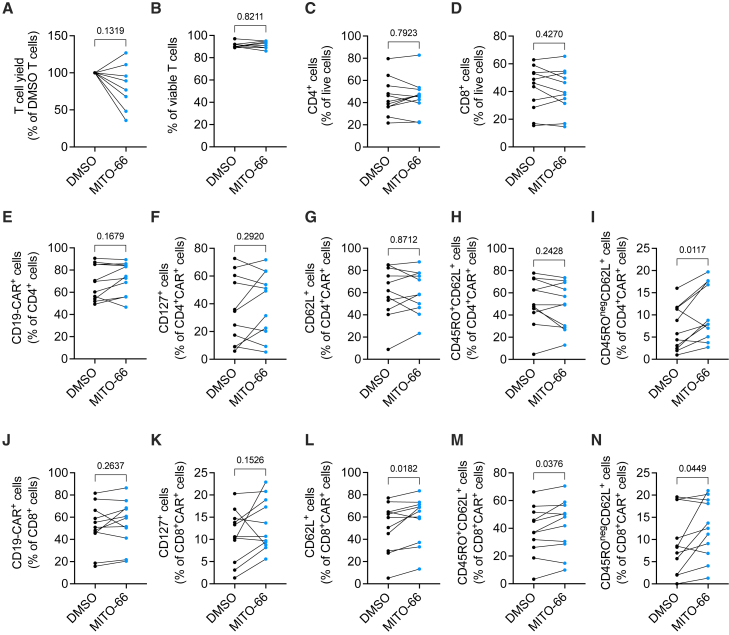
MITO-66 induces a stem cell-like memory phenotype in CAR T cells from patients with B cell malignancies (A) T cell yield at day 10 post-activation, expressed as percentage of DMSO-conditioned CAR T cells. (B) Percentage of viable T cells at day 10 post-activation. (A and B) Eight patients, pooled data from three independent experiments. (C and D) Percentage of CD4 (C) and CD8 (D) T cells in live cells at day 10 post-activation. (E) Percentage of CAR-positive T cells in CD4 T cells at day 10 post-activation. (F–I) Percentage of CD127-positive (F), CD62L-positive (G), CD62L/CD45RO-positive T_CM_ (H), and CD45RO-negative CD62L-positive T_SCM_ (I) cells in CD4 CAR T cells at day 10 post-activation. (J) Percentage of CAR-positive T cells in CD8 T cells at day 10 post-activation. (K–N) Percentage of CD127-positive (K), CD62L-positive (L), CD62L/CD45RO-positive T_CM_ (M), and CD45RO-negative CD62L-positive T_SCM_ (N) cells in CD8 CAR T cells at day 10 post-activation. (C–N) Eleven patients, pooled data from four independent experiments. Data are represented as mean ± SD. Statistics are based on paired, two-tailed Student’s t test.

Healthy donor T cells significantly differ from leukemic patient T cells, which often show signs of exhaustion due to disease state or treatment history.^41^ We therefore generated CD19-CAR T cells in the presence of 25 μM MITO-66 from peripheral blood of a heterogeneous cohort of patients, mainly aged diffuse large B cell lymphoma patients with various treatment histories (Table S1). Similar to healthy donors, presence of MITO-66 during CD19-CAR T cell manufacturing from patient T cells did not affect cell yield, viability, CD4/CD8 ratio, or CAR transduction efficiency (Figures 6A–6E and 6J). MITO-66 induced IL-7 receptor alpha (CD127) expression in both CD4 and CD8 CAR T cells of several, but not all, patients (Figures 6F and 6K). A T_SCM_ phenotype was significantly induced by MITO-66 in both CD4 and CD8 patient CAR T cells, while CD8 CAR T cells also showed an increase in CD62L-positive cells, resulting in a significant increase in T_CM_ cells as well (Figures 6G–6I and 6L–6N).

In summary, MITO-66 treatment during CD19-CAR manufacturing induces a T_SCM_ phenotype in normal as well as in patient T cells.

## Discussion

In this study, we have made use of a novel MPC inhibitor to expand the population of stem cell-like memory CAR T cells during *ex vivo* manufacturing, resulting in a strongly enhanced antitumor efficacy. The development of MITO-66 will not only allow for the application of MPC inhibition in clinical T cell therapy manufacturing *ex vivo*, but could also be used for other therapeutic indications. For example, MPC inhibition has been proposed as a therapeutic strategy against diffuse large B cell lymphoma,^42^ Alzheimer disease,^43^ and alopecia.^44^ In the latter, systemic administration (*in vivo*) would be needed. Despite similarly inducing a T_SCM_ phenotype *in vitro*, and a maintenance of that phenotype in CD8 CAR T cells following *in vivo* transfer, MITO-66-conditioned CAR T cells slightly outperformed UK-5099-conditioned CAR T cells in terms of mouse survival and CAR T cell *in vivo* expansion. Furthermore, UK-5099 had a slightly negative impact on CAR T cell expansion *in vitro* and induced a PD1/TIM3-positive CAR T cell population *in vivo*. These differences could be due to either off-target effects or differences in potency between the two small molecules.

Preclinical and clinical evidence suggests that ACT with memory-enriched T cells results in enhanced therapeutic efficacy.^5^^,^^45^ To this end, numerous strategies have been proposed such as CAR T cell expansion driven by “memory-promoting” cytokines instead of IL-2,^7^^,^^46^ preselection of naive/stem cell-like memory T cells before manufacture,^47^ or reduced manufacture time.^48^^,^^49^ The *ex vivo* use of small molecules is an attractive alternative, as small molecules can be easily integrated and tested in pre-existing cell manufacturing processes. For example, AKTi-conditioned TCR T cells and PI-3Kδ-conditioned CAR T cells have been used in clinical trials (NCT04044859 and NCT03274219, respectively). More recent studies report compelling preclinical data using small molecules against other targets^50^^,^^51^; however, we had to make a limited selection of compounds in order not to end up with an unmanageable number of experimental conditions. Most of the small molecules against which we benchmarked MITO-66 target directly the PI-3Kδ-AKT-mTOR signaling pathways downstream of T cell activation. In contrast, we have previously shown that MPC inhibition enhances chromatin accessibility at pro-memory genes as well as enforces glutamine and fatty acid oxidation to compensate for loss of mitochondrial pyruvate import.^19^ By differently affecting mitochondrial metabolism, IDH2 inhibition also enhances glutamine and fatty acid oxidation,^15^ suggesting that these metabolic alterations and their epigenetic crosstalk might be key to the superior antitumor efficacy we observed in our benchmark study.

In conclusion, steering cellular metabolism during CAR T cell manufacturing by inhibiting the MPC with MITO-66 induces a T_SCM_ phenotype that translates into potent *in vivo* antitumor efficacy. Although we demonstrated that MITO-66 can induce a T_SCM_ phenotype in T cells derived from patients with B cell malignancies, compatibility with a clinical-grade process, including media and culture devices, would still need to be shown before successful clinical translation.

## Materials and methods

### Mice, cell lines, human samples

NSG mice were purchased from The Jackson Laboratory. Males and females between 6 and 10 weeks old were used for experiments. Sample size was chosen based on previous experience. Researchers were not blinded to the different treatment groups. Mice were kept in the animal facility of Agora in Lausanne in individually ventilated cages, between 19°C and 23°C with 45%–65% humidity and a 12-h dark/light cycle. Experimentation was performed respecting the protocols approved by the veterinary authorities of the Canton de Vaud (VD3763b).

HEK293T, NALM6, and T2 cells were obtained from the American Type Culture Collection and were not further authenticated. All cell lines were cultured in RPMI, containing 10% fetal bovine serum (FBS), 1% penicillin/streptomycin (P/S). Polymerase chain reaction testing was systematically done to confirm that cell lines were mycoplasma free.

Peripheral blood from de-identified healthy human volunteers was obtained from the Center of Interregional Blood Transfusion SRK Bern. Peripheral blood mononuclear cells were obtained from patients with B cell malignancies (Table S1) at the Geneva University Hospital under a research protocol approved by the cantonal ethical commission for human research (CCER-2023-02042), and after obtaining informed consent.

### MITO-66 IC_50_ determination

#### BRET

BRET analyses were performed as described previously,^27^ using HEK293T cells stably transfected with MPC1-Rluc8 and MPC2-Venus. Cells were maintained in DMEM, 10% FBS, 1% P/S, 2 mM glutamine. For the assay, cells were resuspended at 10^6^ cells/mL and 100 μL seeded in white 96-well plates (Greiner) and grown overnight. The cells were carefully washed in PBS, 1 mM CaCl_2_, and 0.5 mM MgCl_2_ and the assay was performed in the same buffer supplemented with 5 μM coelenterazine h, the luminescent substrate for Rluc8. The test compounds, typically a 1:3 dilution series starting at 30 μM were added, and compound-induced changes in luminescence intensity were measured over time in a Synergy 2 plate reader (BioTek). BRET values were quantified as described previously.^27^

#### Seahorse determination of oxygen consumption rate

Mito stress test assays were performed in the Seahorse XFe24 Flux Analyzer (Seahorse Biosciences) using the protocols provided by the manufacturer. In brief, HeLa cells were resuspended at 2 × 0^5^/mL and 200 μL aliquots added to each well. Cells were allowed to settle undisturbed to ensure uniform distribution, then incubated overnight at 37°C, 5% CO_2_. For the assay, cells were washed twice in the assay medium, PBS supplemented with 1 mM CaCl_2_, 0.5 mM MgCl_2_, 1 mM pyruvate. After washing, 450 μL fresh assay buffer was added to each well and the plate was transferred to the XFe24 Flux Analyser, in which the injection ports had been primed with 10× reagents as follows: port A: different MPC inhibitors; port B: 20 μM oligomycin A; port C: 10 μM FCCP; port D: 10 μM rotenone +10 μM antimycin. Compound potency was determined from the data following injection of the uncoupler FCCP, during which the maximal rate of oxygen consumption is measured.

### Thermoshift analysis

Stock solution of purified MPC1/MPC2 was incubated with a serial dilution of MITO-66 and resulting protein concentration was 11 μM. For the measurement, a nanoDSF Prometheus NT.48 device from NanoTemper Technologies (München, Germany) with Prometheus High Sensitivity Capillaries was used. Excitation LED power was set to 100%, and fluorescence data were collected in the temperature range from 15°C to 80°C with temperature ramp of 1°C/min.

Estimation of binding affinity is based on the analysis of melting temperature shift in the presence of MITO-66. Analysis was done with help of an eSPC online data-analysis platform,^52^ based on the FoldAffinity method.^53^ Fluorescence ratio 350 nm/330 nm was fit in the temperature range of 20°C–70°C. Estimation of Kd was done by fitting a model with a single binding site in the temperature range of 43°C–47°C, with best fits being at 44.3°C. The confidence interval (marginal asymmetric confidence interval at a 95% confidence level) was estimated as suggested by Paketuryte et al.^54^

### Human anti-CD19 CAR T cell preparation

The hCD19-28z CAR was constructed by ligating the hCD19 scFv (FMC63) into the CAR backbone sequences of the third-generation viral vector pTRPE-28z. Third-generation lentiviral vectors were produced as described previously.^55^ In brief, 95% confluent HEK293T cells were transfected with pTRPE, psPAX2 encoding gag-pol, and pMD2.G encoding the VSV-G envelope, using Lipofectamine 2000 (Invitrogen). Viral supernatant was collected 24 and 48 h post-transfection, filtered, concentrated by ultracentrifugation, and frozen. Viral titer was determined by serial dilution transfection in T2 cells for 72 h, followed by flow cytometry-based determination of transduction efficiency. For the generation of CAR T cells from healthy donors, T cells were enriched from peripheral blood by RosetteSep (STEMCELL Technologies). For the generation of CAR T cells from patient samples, whole peripheral blood mononuclear cells were cultured at 10^5^ cells per 100 μL in round-bottom 96-well plates. T cells were activated with human anti-CD3/CD28 Dynabeads (Gibco) and cultured in RPMI supplemented with 10% FBS, 1 mM HEPES, 1% P/S, and 1% non-essential amino acids (R10, all Gibco). Twenty four hours later, T cells were transduced at an MOI of 3 with the lentiviral vector encoding anti-human CD19scFv fused to CAR backbones containing human CD28 and CD3ζ(CD247) signaling domains and expanded *ex vivo* for 10–12 days. Transduced T cells were maintained at a concentration of 0.75 × 10^6^ cells/mL throughout the culture period by cell enumeration every 2–3 days. T cells were exposed to the indicated concentrations of small-molecule inhibitors or solvent only (DMSO) throughout the entire culture period. At the end of the expansion, T cells were washed and frozen in 90% FBS/10% DMSO and stored in a liquid nitrogen tank.

### *In vitro* killing

Frozen CAR T cells were thawed and washed with R10. CAR T cells were counted and 10^5^ live CAR T cells were incubated with 2 × 10^5^ NALM6 cells in a 96-well plate. Triplicate wells were harvested at 72 h and after 7 days for flow cytometry-based quantification of NALM6, CD4, and CD8 CAR T cells by using precision counting beads (BioLegend). Duplicate wells were incubated with 5 μg/mL Brefeldin A (BioLegend) after 67 h of co-culture to allow for intracellular accumulation of cytokines. Five hours later, wells were harvested for intracellular cytokine staining and flow cytometry analysis.

### B-ALL xenograft model

NSG mice were inoculated with 10^6^ NALM6 cells in the tail vein. Fourteen or 15 days after NALM6 infusion, human CAR T cells were thawed, washed and 2 × 10^6^ CAR^+^ cells were adoptively transferred in the tail vein. Body weight and health of the mice were regularly monitored. When the physical state and behavior of mice declined below the levels established by the Swiss cantonal authorities, or body weight decreased by more than 15%, mice were sacrificed. NALM6 cell numbers in the blood were measured by anti-human CD19 flow cytometry analysis.

### B-ALL xenograft *in vivo* serial rechallenge

Luciferase-expressing NALM6 (NALM6-Luc) cells were generated by lentiviral transduction of NALM6 cells with the lentiviral vector “Lenti-luciferase-P2A-Neo,” a gift from Christopher Vakoc^56^ (Addgene, no. 105621; http://n2t.net/addgene:105621;RRID:Addgene_105621). NALM6-Luc (10^6^) was intravenously injected in NSG mice. Seven days later, 3 × 10^6^ DMSO or MITO-66-conditioned CD19-CAR T cells were transferred in the tail vein. Blood was sampled at the indicated time points for flow cytometric analysis. Tumor growth was monitored by bioluminescence imaging using the Xenogen *in vivo* imaging system (Caliper Life Sciences), following intraperitoneal injection of 150 mg/kg D-Luciferin (Promega).

### Flow cytometry

Fluorochrome-conjugated antibodies were all from BioLegend or BD Biosciences. Staining for flow cytometry was done in PBS with 2% FBS and 2 mM EDTA, at 4°C in the dark. The Live/Dead Fixable yellow Cell Stain Kit (Thermo Fisher Scientific) was used to mark dead cells. CD19-CAR T cells were stained with recombinant human Alexa Fluor 647-tagged CD19 protein (R&D Systems).

Intracellular staining for IFN-γ was performed by fixing and permeabilizing the cells with a BioLegend kit and an FITC-labeled anti-IFN-γ antibody.

For the flow cytometry-based measurement of fatty acid and amino acid oxidation, we applied the SCENITH method as described previously.^57^ In brief, 150,000 T cells were seeded in 96-well plates and incubated for 45 min with control, 2-DG (100 mM), oligomyin (1 mM), or a combination of 2-DG and oligomycin. During the last 15 min of this incubation, puromycin was added at a final concentration of 10 mg/mL. Cells were then washed with PBS, stained with the Live/Dead kit followed by surface marker staining. Next, intracellular staining of puromycin was performed by using the FOXP3 staining kit buffers (Thermo Fisher Scientific) and a PE-labeled anti-puromycin monoclonal antibody (BioLegend).

Acquisition was done on Fortessa and Symphony flow cytometers with FACSDiva Software (BD Biosciences).

The flow cytometry gating strategy for T_SCM_ cells is illustrated in Figure S4.

### Western blot

For histone analyses, nuclei were isolated and washed in Triton extraction buffer (PBS containing 0.5% Triton X-100) containing 5 mM sodium butyrate and 500 μM protease inhibitor, and histones were acid extracted with 0.2 M HCl, followed by precipitation with trichloroacetic acid. Histones were then resuspended in 50 mM Tris-HCl. Histone extracts were quantified with Bradford reagent (Sigma-Aldrich). Equal amounts of proteins were denatured for 5 min at 95°C in SDS loading dye containing 5% β-mercaptoethanol, followed by separation on 4%–12% Bis-Tris Plus gradient gel (Invitrogen) and transferring onto 0.2 μM nitrocellulose membranes (Bio-Rad). After transfer, blocking of unspecific binding sites was done in 5% milk and incubation with primary antibodies (Cell Signaling Technology) was done overnight at 4°C. The next day, membranes were incubated with HRP-conjugated secondary anti-rabbit antibodies (Santa Cruz Biotechnology). Chemiluminescence imaging was performed with ECL and Femto reagents (Super Signal West, Thermo Fisher Scientific).

### Quantification and statistical analysis

FlowJo v.10 was used to analyze flow cytometry data. Prism v.9 software (GraphPad) was used for statistical analyses. Results are represented as mean ± standard deviation (SD). Information on each statistical test used is provided in figure legends, with sample size and number of independent repeats. Two group comparisons were tested by unpaired two-tailed Student’s t tests. Comparisons of more than two groups were done with one-way ANOVA. Grouped data comparisons were calculated using two-way ANOVA. Graft-versus-host disease or death led to the exclusion of some mice from analyses. Samples were excluded from flow cytometry analyses when the number of events recorded was lower than 20 in the population of interest, preventing accurate analyses.

## Data and code availability

For detailed protocols and all data not publically accessible, request can be made to mathias.wenes@unige.ch and denis.migliorini@unige.ch.

## Acknowledgments

We would like to thank the scientific platforms at AGORA, Lausanne: the In Vivo Center, the Flow Cytometry Facility, and the In Vivo Imaging Facility. We also thank the Protein Production and Structure Core Facility at EPFL for the adaptation and production of the MPC proteins, especially Laurence Durrer and Soraya Quinche. The graphical abstract was generated in BioRender. P.R. was supported in part by a grant from the 10.13039/501100004361Swiss Cancer League, no. KFS-4404-02-2018. D.M. was supported by the Swiss Institute for Experimental Cancer Research – 10.13039/501100017035ISREC Foundation, and the 10.13039/501100013348Swiss Innovation Agency (Innosuisse) for this project.

## Author contributions

M.W. conceived, designed, and performed most experiments and wrote the manuscript. A.L. designed and performed the experiments. V.A. and O.B. performed thermoshift assay. K.M. performed BRET and Seahorse experiments. F.S. and V.D provided PBMCs from patients. P.R. gave scientific input and provided overall direction. J.-C.M. gave scientific input and developed MITO-66. D.M. gave scientific input, designed experiments, and provided overall direction. All co-authors reviewed and edited the manuscript.

## Declaration of interests

M.W. and P.R. are inventors on a patent on MPC inhibition for memory T cell development. M.W. and J.-C.M. are part of the management team at MPC Therapeutics, a Geneva-based start-up that develops MITO-66. D.M. and P.R. are members of the scientific advisory board at MPC Therapeutics. D.M. is an inventor of patents related to CAR-T cell therapy, filed by the University of Pennsylvania, the Istituto Oncologico della Svizzera Italiana (IOSI), and the University of Geneva. D.M. is a scientific cofounder of Cellula Therapeutics SA.

## Supplemental information

Document S1. Figures S1–S4 and Table S1

Document S2. Article plus supplemental information
