# Supplementary material for: A novel mitochondrial pyruvate carrier inhibitor drives stem cell-like memory CAR T cell generation and enhances antitumor efficacy
Source: Mol Ther Oncol. 2024 Oct 18;32(4):200897. doi: 10.1016/j.omton.2024.200897 (PMC11570499; doi:10.1016/j.omton.2024.200897)
Supplement: Document S1. Figures S1–S4 and Table S1 [file mmc1.pdf]

OMTON, Volume *32*

Supplemental information

A novel mitochondrial pyruvate carrier inhibitor drives stem cell-like memory CAR T cell generation and enhances antitumor efficacy

Mathias Wenes, Anouk Lepez, Vladimir Arinkin, Kinsey Maundrell, Orsolya Barabas, Federico Simonetta, Valérie Dutoit, Pedro Romero, Jean-Claude Martinou, and Denis Migliorini

Supplemental Material

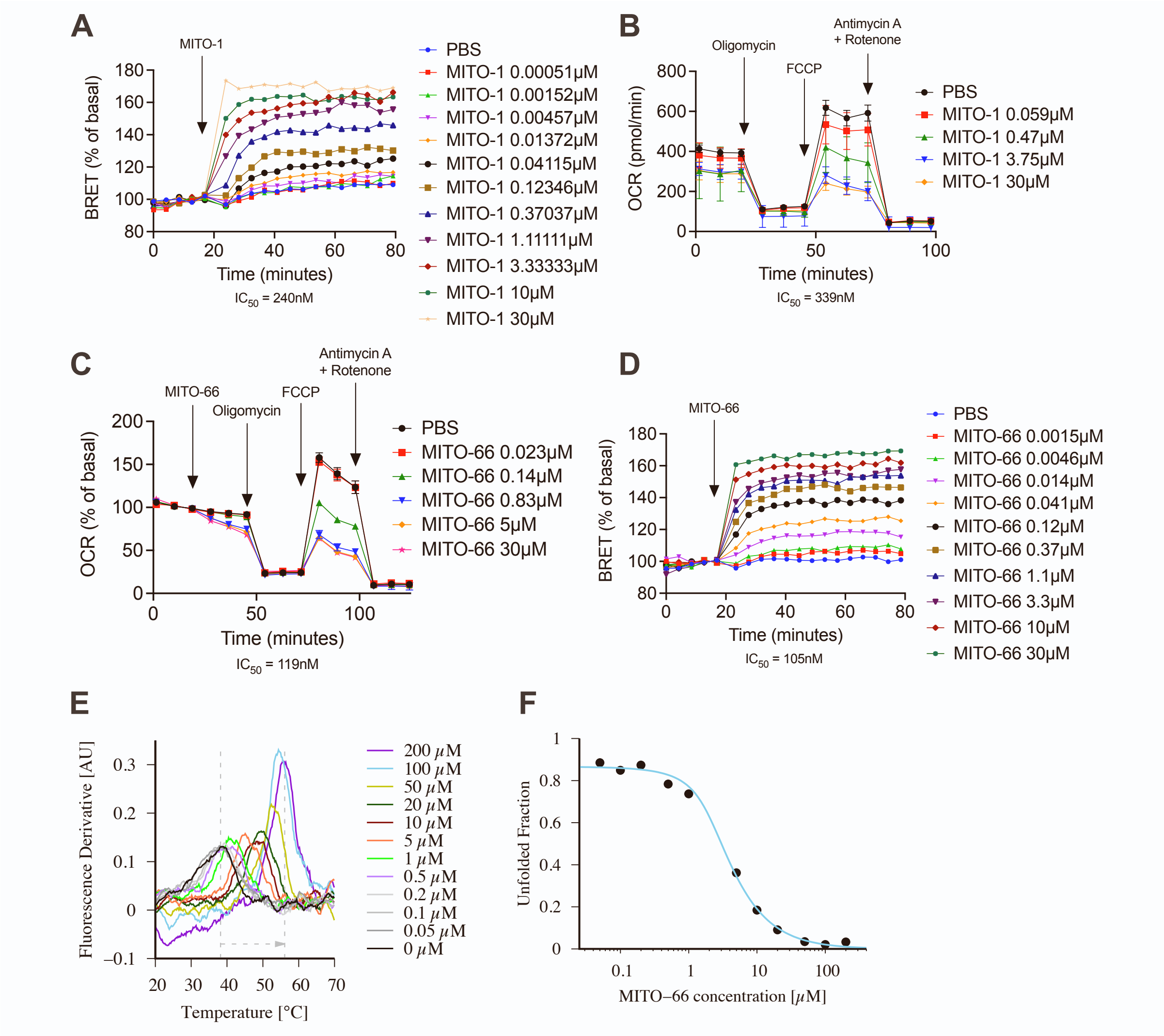

Figure S1. MITO-66 is a novel small molecule inhibitor of the mitochondrial pyruvate carrier.

(**A**) BRET assay in HEK293T cells with MITO-1 or PBS as control.

(**B-C**) Oxygen consumption rate (OCR) in HeLa cells measured by Seahorse using MPC inhibitor MITO-1 (B) or MITO-66 (C). IC_{50} was calculated based on drop in maximal oxygen consumption rate.

(**D**) BRET assay in HEK293T cells with MITO-66 or PBS as control.

(**E**) NanoDSF thermoshift analysis shows MPC1/MPC2 heterocomplex stability induced by MITO-66 binding. Fluorescence-based melting curves at different concentrations of MITO-66.

(**F**) Isothermal analysis for Kd estimation at 44.3°C (6.1°C above the protein Tm).

Data is represented as mean ± standard deviation (SD).

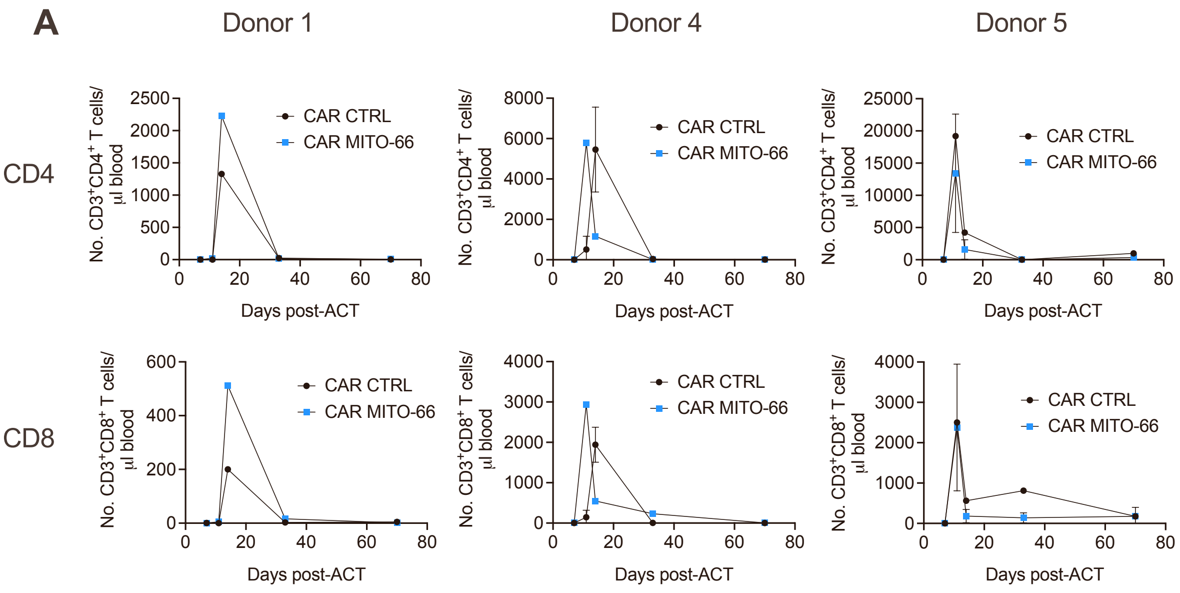

Figure S2. MITO-66 conditioning during CAR T cell manufacturing enhances anti-tumor efficacy

(**A**) Number of transferred CD4 or CD8 T cells in the blood of mice, derived from donors that induced survival following either DMSO- and MITO-66 conditioning.

Data is represented as mean ± SD.

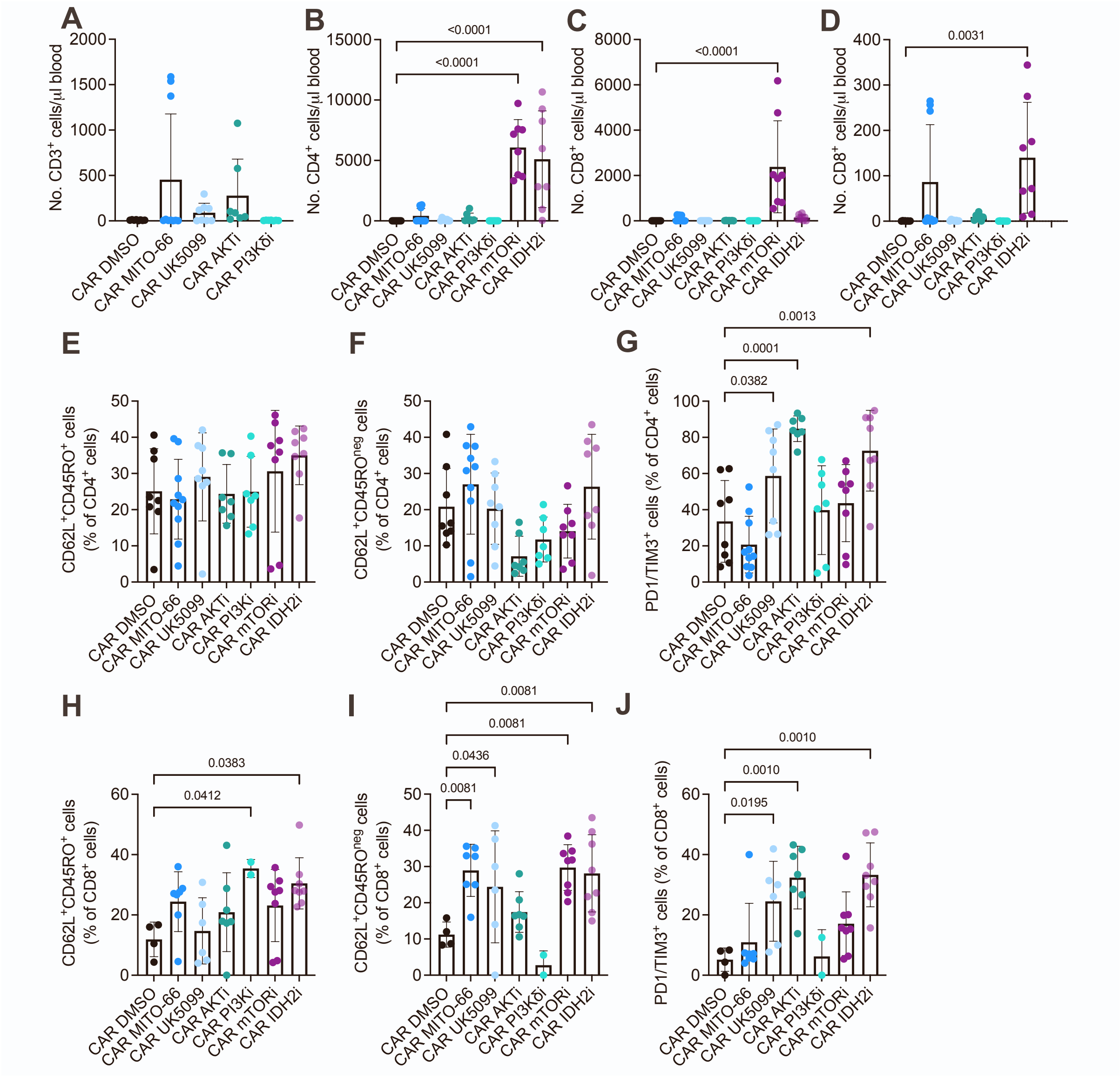

Figure S3. Benchmarking MITO-66 against other small molecules influencing memory differentation

(**A**) Number of transferred CD3 T cells in the blood of mice analysed by flow cytometry at day 11 post-ACT. Similar data as main Figure 5H, graph without rapamycin- and IDH2i-CAR T cells for clarity.

(**B-C**) Number of transferred CD4 (B) or CD8 (C) T cells in the blood of mice analysed by flow cytometry at day 11 post-ACT.

(**D**) Number of transferred CD8 T cells in the blood of mice analysed by flow cytometry at day 11 post-ACT. Similar data as Supplemental Figure 3C, graph without rapamycin-CAR T cells for clarity.

(**E-G**) Percentage of CD62L/CD45RO double positive cells (E), CD62L-positive, CD45RO-negative cells (F) or PD1/TIM3 double positive cells (G) in transferred CD4 T cells at day 11 post-ACT.

(**H-J**) Percentage of CD62L/CD45RO double positive cells (H), CD62L-positive, CD45RO-negative cells (I) or PD1/TIM3 double positive cells (J) in transferred CD8 T cells at day 11 post-ACT. (A-J: 3 human donors into 9-10 total mice, pooled data from 2 independent experiments. Only flow cytometry data with >20 events were used for phenotypic analysis).

Data is represented as mean ± SD. Statistics are based on one-way ANOVA.

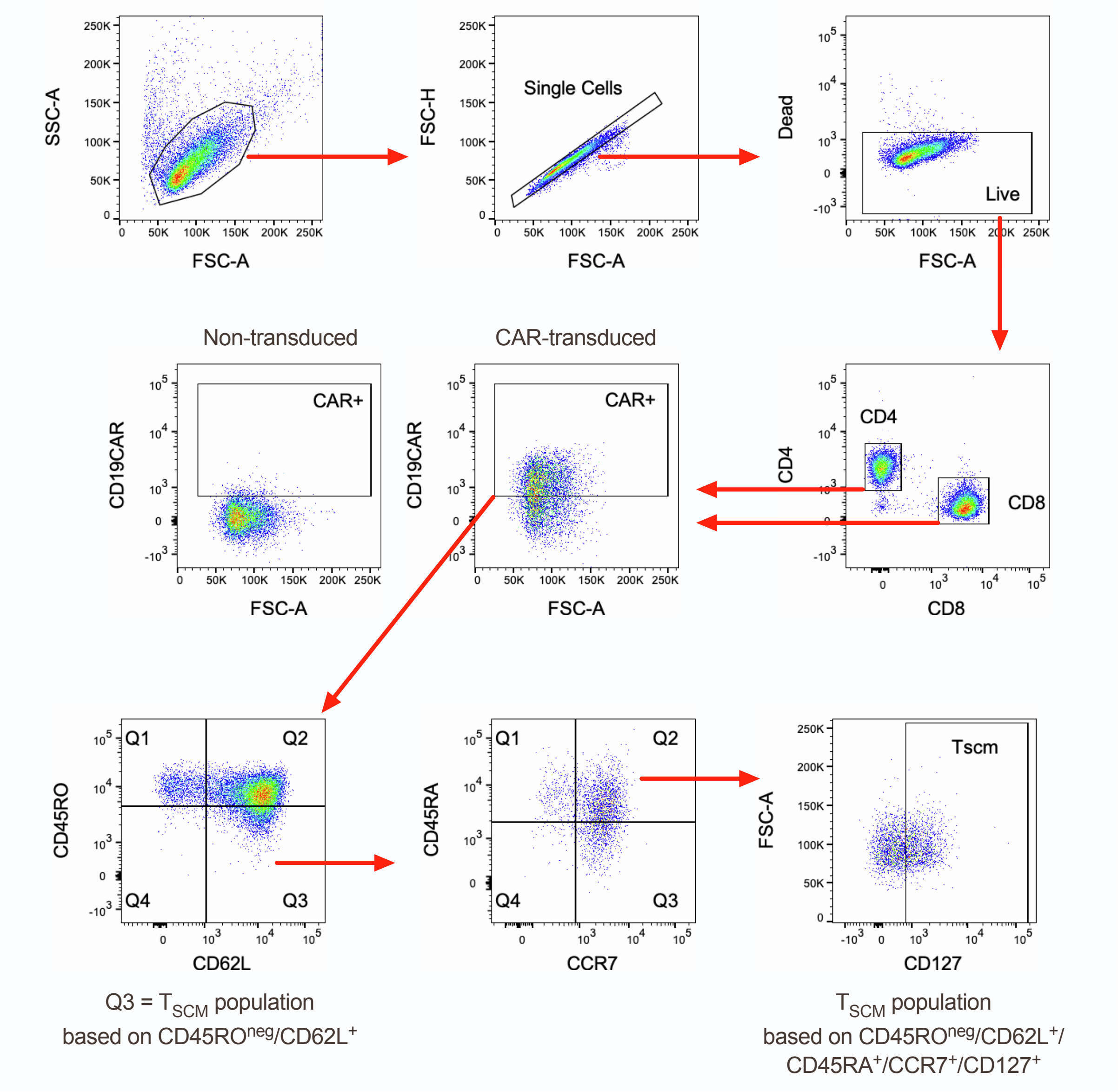

Figure S4. Flow cytometry gating strategy

Strategy used for selecting live, single cells for further downstream division in memory T cell subsets.

Table S1: Clinical parameters of patient samples used for experiments in Figure 6.

Patient	Sex	Age at Diagnosis	Diagnosis	Treatment received before sample collection
1	M	64	DLBCL	Methotrexate, CCNU, procarbazine, steroids
2	F	75	DLBCL	No
3	F	60	DLBCL	No
4	M	76	DLBCL	No
5	M	77	Follicular lymphoma	No
6	M	68	DLBCL	No
7	M	71	DLBCL	R-CHOP, R-ICE
8	M	57	Richter's Syndrome	R-CHOP, R-DHAP, Ibrutinib, allogeneic HSCT
9	F	69	DLBCL (Transformed follicular lymphoma)	R-Bendamustine, R-CHOP
10	F	71	DLBCL	R-CHOP, MATRIx
11	M	58	DLBCL	R-CHOP, R-ICE
